# Supplementary material for: Use of Pseudomonas protegens to Control Root Rot Disease Caused by Boeremia exigua var. exigua in Industrial Chicory (Cichorium intybus var. sativum Bisch.)
Source: Plants (Basel). 2024 Jan 17;13(2):263. doi: 10.3390/plants13020263 (PMC10818772; doi:10.3390/plants13020263)

**Supplementary material S1.**

**Visual scale used to rate industrial chicory root rot in the field.**

Modified 7-level scale for sugar beet root rot described by Hanson (2010) and used by Beneo Orafti S.A. and in this study to assess root rot in industrial chicory in the field, where 0= No rot symptoms; 1 = With surface rot spots; 2 = With rot band of < 5mm penetration; 3.1 = With less than 5 mm penetration into the rot band and < 30% root rot; 3.2 = With more than 5 mm of penetration in the rot band and > 30% rot; 4.1= Root tip rot and advance < 5 cm from apex, 4.2= Advanced tip rot > 5 cm advance from apex and 5= 100% completely rotten root.

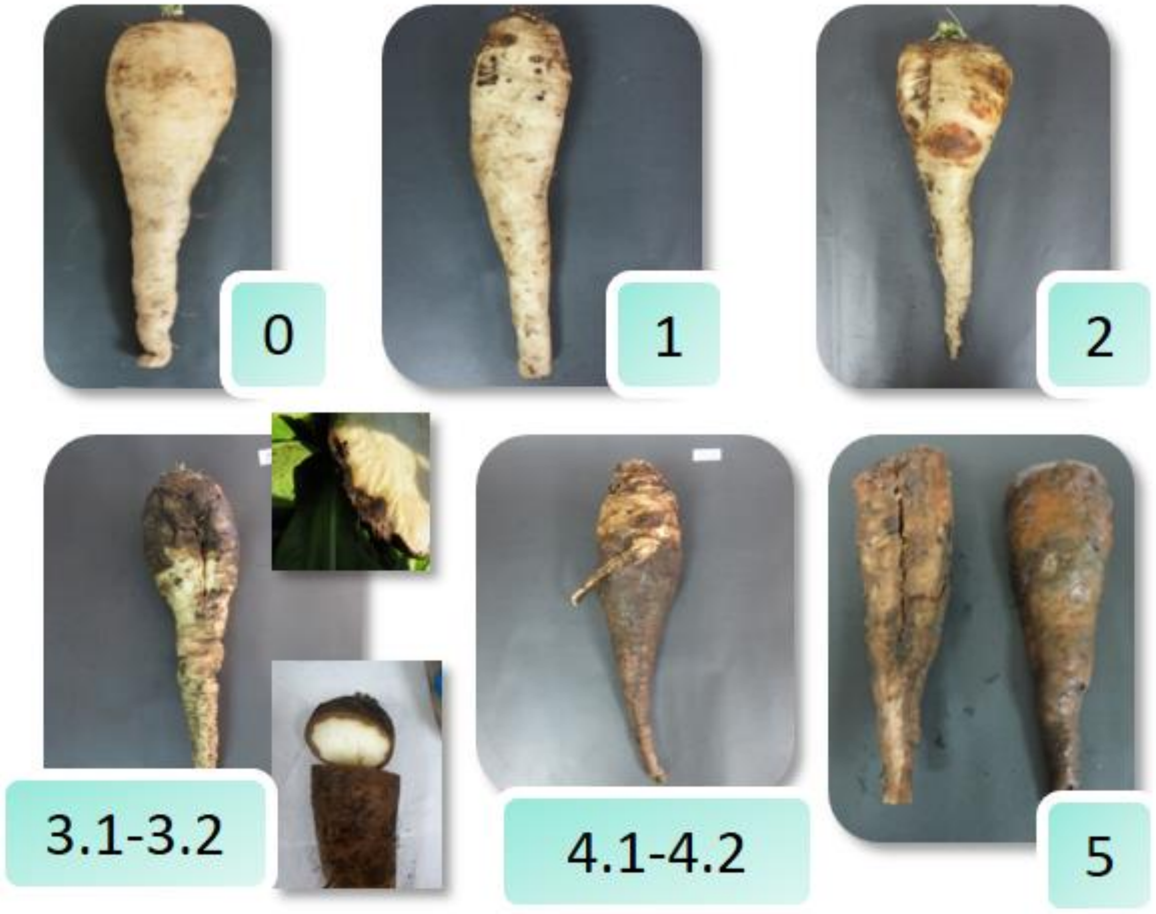

**Field assessments for the presence of the *phlD* gene in bacterial populations in the roots of chicory plants at different growth stages (days after seeding, DAS)**

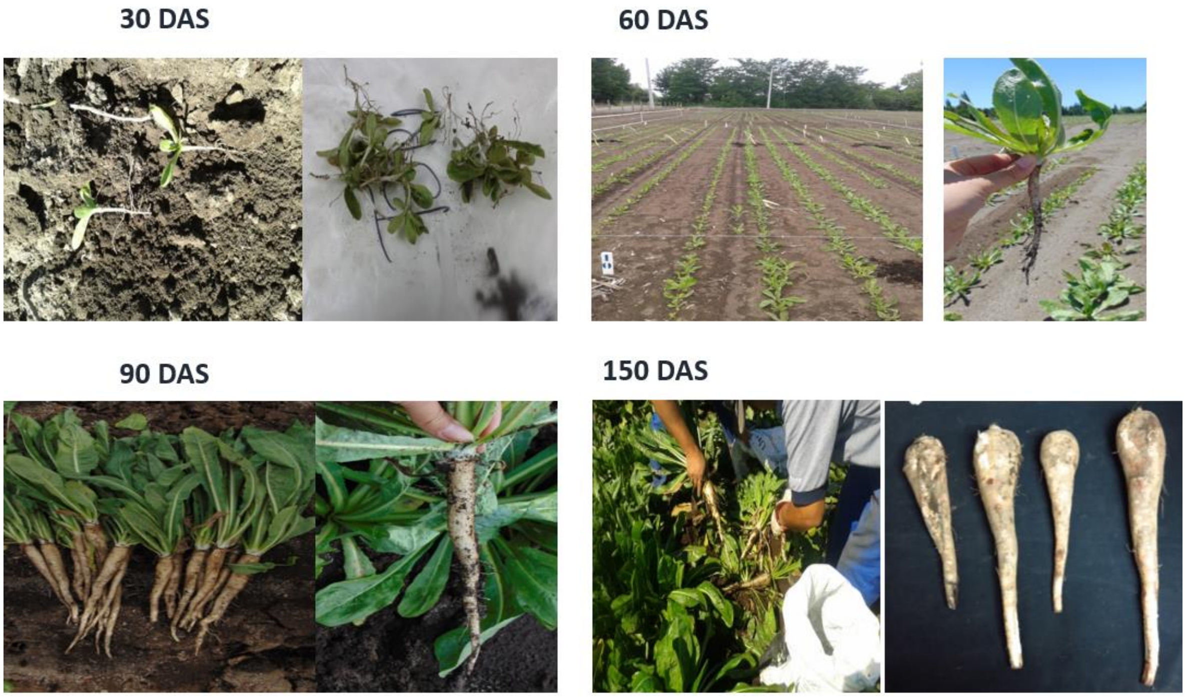

**Steps of measurement of *Pseudomonas protegens* population by using the *phlD*-specific PCR based dilution endpoint assay.**

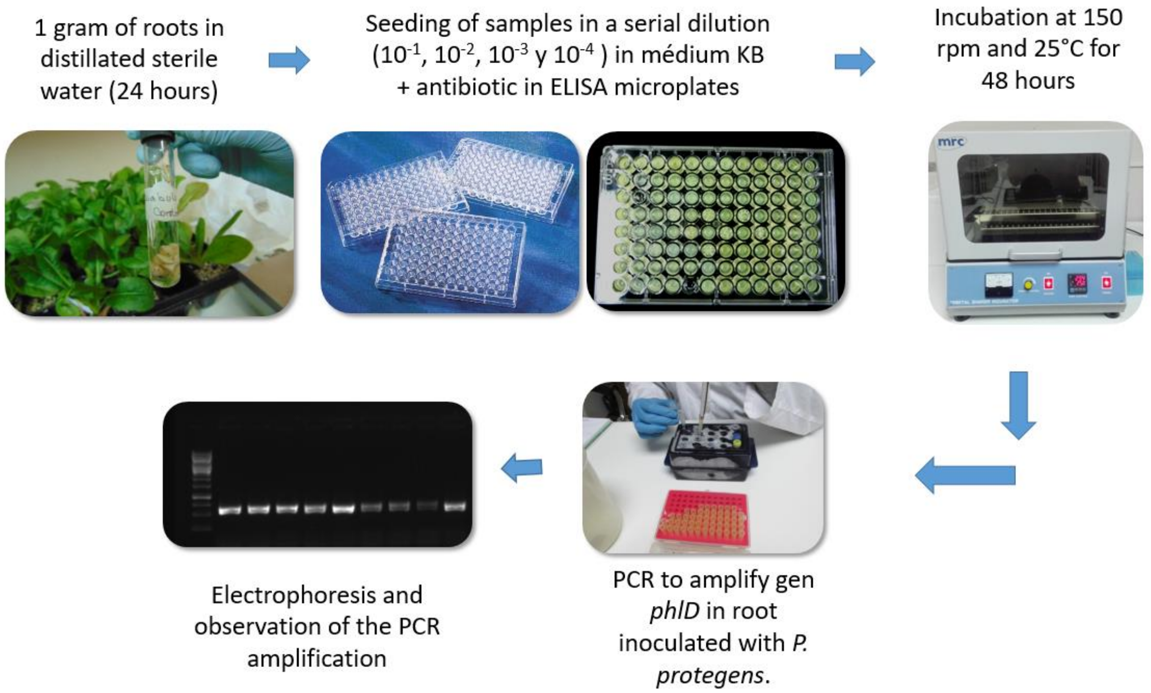


**View of an electrophoresis gel to assess *Pseudomonas protegens* populations by using the *phlD*-specific PCR based dilution endpoint assay.**

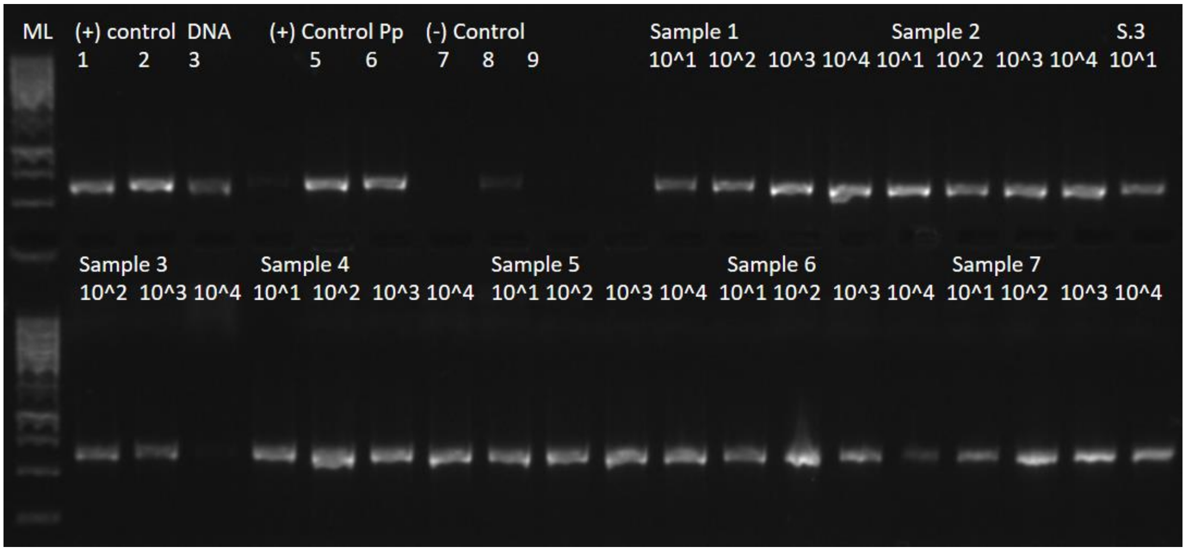

Supplement: Supplementary file 1 [file plants-13-00263-s001.zip › Supplementary material S1 Root rot scale.pdf]
