# Supplementary material for: Use of Pseudomonas protegens to Control Root Rot Disease Caused by Boeremia exigua var. exigua in Industrial Chicory (Cichorium intybus var. sativum Bisch.)
Source: Plants (Basel). 2024 Jan 17;13(2):263. doi: 10.3390/plants13020263 (PMC10818772; doi:10.3390/plants13020263)

Supplementary material S2. View of results of in vitro tests

In vitro preselection of the antagonistic effect of bacterial isolates on the pathogenic fungus *Boeremia exigua* var. *exigua*.

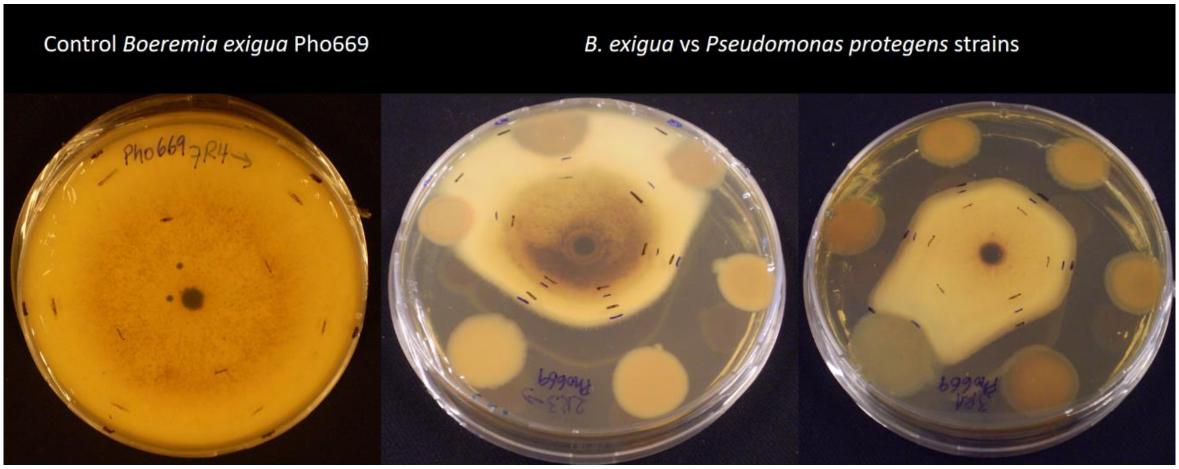

Mycelial growth inhibition observed on *Boeremia exigua* var. *exigua* strain Pho669 obtained in dual cultures by two strains of *Pseudomonas protegens* bacteria after 14 days under in vitro conditions.

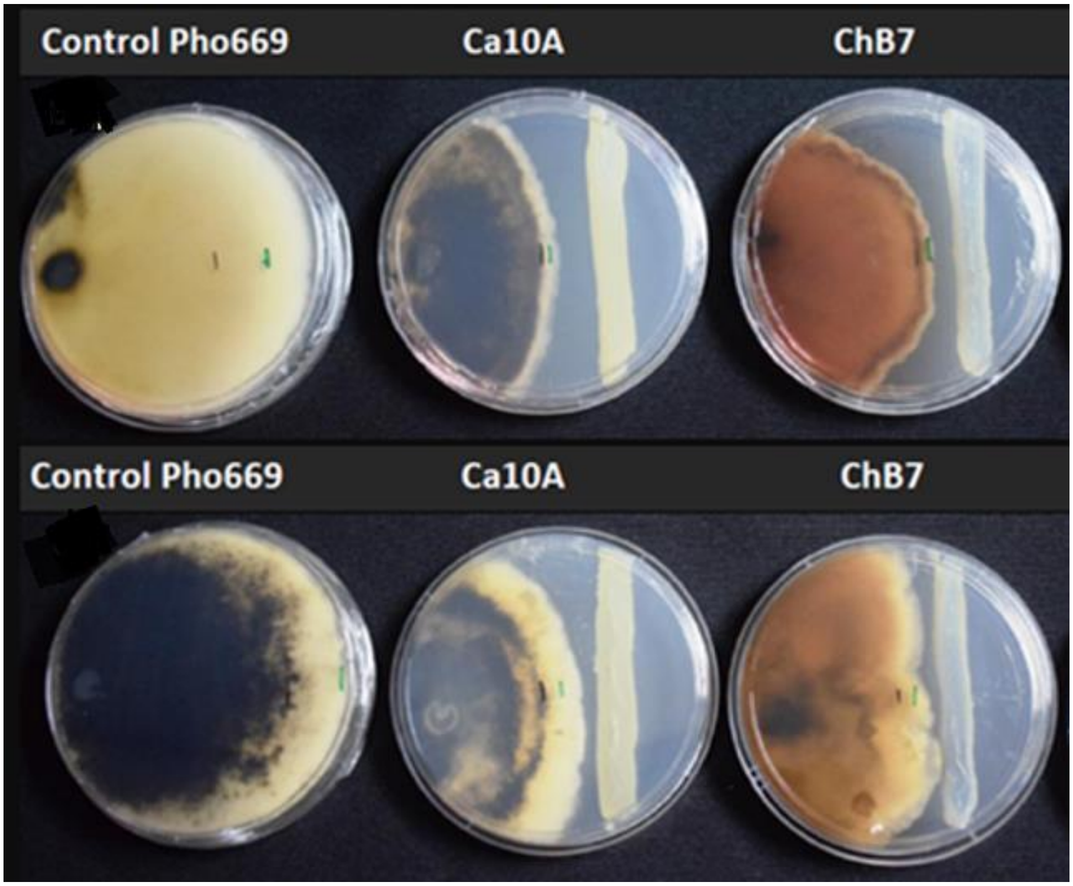

Panoramic view of the Selva Negra Experimental Station (A) and an inoculated plot with affected plants by *Boeremia exigua* var. *exigua* (B). Healthy chicory roots and other affected by *B. exigua* var. *exigua* observed in the laboratory (C). Chicory root with typical symptoms caused by *B. exigua* var. *exigua* in the field (D)

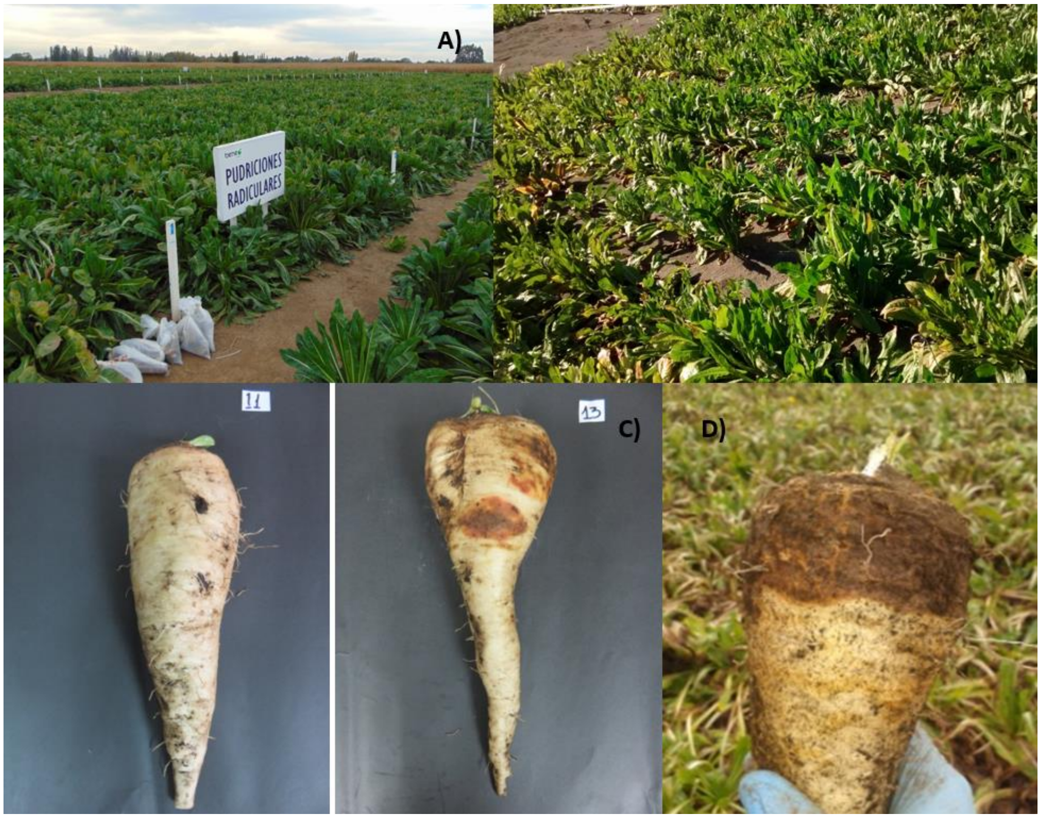

Symptoms on the roots (left) and view of the necrotic penetration band caused by *Boeremia exigua* var. *exigua* inside the chicory roots (right).

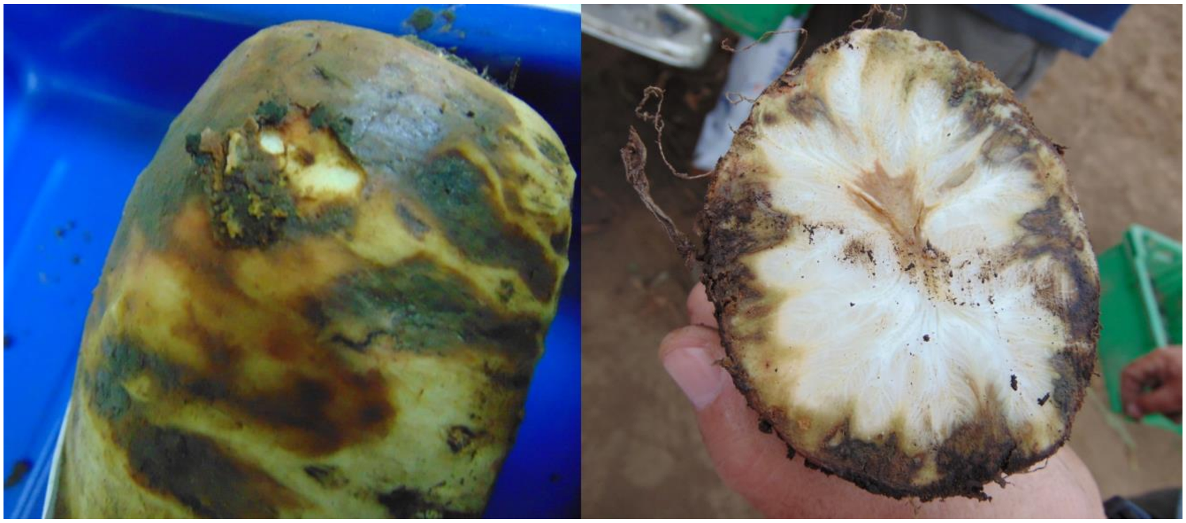

Supplement: Supplementary file 1 [file plants-13-00263-s001.zip › Supplementary material S2 View in vitro tests.pdf]
