# Supplementary material for: Use of Pseudomonas protegens to Control Root Rot Disease Caused by Boeremia exigua var. exigua in Industrial Chicory (Cichorium intybus var. sativum Bisch.)
Source: Plants (Basel). 2024 Jan 17;13(2):263. doi: 10.3390/plants13020263 (PMC10818772; doi:10.3390/plants13020263)
Supplement: Supplementary file 1 [file plants-13-00263-s001.zip › Supplementary material S3 Agrometeorological condition of field experiments.pdf]

Supplemenatary material S3. Agrometeorological conditions observed in the field experiments.

Agrometeorological data during the 2015-16 industrial chicory season, obtained from the Santa Rosa Agrometeorological Station, Chillán, Ñuble Region, Chile. Data obtained from the National Agroclimatic Network (Agromet), online.

Month	Rain accumulation (mm)	Evapotranspiration (mm)	Air average temperature (°C)	Soil average temperature (°C)
**September**	103.8	56.4	10.4	10.4
**October**	101.5	90.2	13.0	12.9
**November**	8.7	120.3	16.0	15.6
**December**	0.0	158.4	19.0	18.1
**January**	8.6	150.2	21.4	19.6
**February**	0.0	141.6	20.1	18.8
**March**	6.6	103.9	17.4	16.4
**April**	104.8	47.6	12.9	13.6
**May**	55.6	27.9	11.9	12.8

Agrometeorological data during the 2015-16 industrial chicory season, obtained from the Human Agrometeorological Station, Quilleco, Biobío Region, Chile. Data obtained from the National Agroclimatic Network (Agromet), online.

Month	Rain accumulation (mm)	Evapotranspiration (mm)	Air average temperature (°C)	Soil average temperature (°C)
**October**	93.2	95.4	13.1	13.1
**November**	22.4	120.7	13.9	16.1
**December**	6.4	160.1	19.1	18.7
**January**	0.9	160.4	21.4	22.5
**February**	0.2	145.8	21.1	21.5
**March**	20.1	117.8	17.9	18.7
**April**	109.8	51.7	12.2	12.6
**May**	40.7	29.1	10.7	11.7
